# Transcriptome-based analysis of resistance mechanism to black point caused by *Bipolaris sorokiniana* in wheat

**DOI:** 10.1038/s41598-021-86303-1

**Published:** 2021-03-25

**Authors:** Qiaoyun Li, Chuang Gao, Kaige Xu, Yumei Jiang, Jishan Niu, Guihong Yin, Chenyang Wang

**Affiliations:** Agronomy College/National Key Laboratory of Wheat and Maize Crop Science, Henan Agricultural University, No. 15 Longzi Lake University Zone, New East District of Zhengzhou, Zhengzhou, 450046 Henan People’s Republic of China

**Keywords:** Transcriptomics, Plant stress responses

## Abstract

Black point is a cereal disease caused by complex pathogens, of which the pathogenicity of *Bipolaris sorokiniana* is the most serious in wheat. Resistance to black point is quantitative in nature, and thus the mechanism is poorly understood. We conducted a comparative transcriptome analysis to identify differentially expressed genes (DEGs) in black point-slightly susceptible and -highly susceptible wheat lines at different timepoints following *B. sorokiniana* inoculation. DEGs associated with photosynthesis were upregulated in black point-slightly susceptible lines. The top Gene Ontology enrichment terms for biological processes were oxidation–reduction, response to cold, salt stress, oxidative stress, and cadmium ion; terms for cellular component genes were mainly involved in plasma membrane and cytoplasmic membrane-bounded vesicle, whereas those for molecular function were heme binding and peroxidase activity. Moreover, activities of antioxidant enzymes superoxide dismutase, catalase, and peroxidase were higher in slightly susceptible lines than those in highly susceptible lines (except peroxidase 12–24 days post-inoculation). Thus, resistance to *B. sorokiniana*-caused black point in wheat was mainly related to counteracting oxidative stress, although the specific metabolic pathways require further study. This study presents new insights for understanding resistance mechanisms of selected wheat lines to black point.

## Introduction

Black point disease is common in wheat (*Triticum aestivum* L.) grown throughout the world and is characterized by a dark or brown discoloration of kernel embryos. In severe cases, the discoloration covers the whole kernel and causes shrinkage^1–4^. Black point results in economic losses due to a reduction in the commercial grade of the grain from infected seeds with a discolored appearance^5,6^ and also affects the effectiveness of wheat seeds as infection decreases seed germination, inhibits seedling growth, and reduces grain yield^3,7^. This disease can also lead to a serious food safety problem because some of the infecting fungal species that cause black point may produce toxic substances^8,9^.

The incidence of black point has attracted research attention from a range of countries^1,3–7,9,10^. Although the damage caused by black point can be reduced by the application of chemical and biological agent^3^, the best strategy to control this disease is by planting more resistant wheat cultivars. However, resistance may be limited; for instance, in the North China Plain, more than 60% of 403 wheat genotypes were identified as being susceptible to black point from 2010 to 2012^7^.

Understanding the mechanism of resistance to black point is critical for breeding disease-resistant and high-yielding cultivars. Currently, research on black point has focused mainly on the damage^5–7^, causative pathogens^6,11,12^, influencing factors^13,14^, and control methods^2,3^ and also in quantitative trait locus/genes mapping for disease resistance^15–20^, although there are few studies on resistance mechanisms.

In wheat, resistance to black point is quantitative in nature and the underlying genetic factors are also highly complex, and experimental errors may have previously masked differences in resistance levels among genotypes^4,16,20^. To date, 230 loci involved in resistance to black point have been reported and are located throughout the wheat genome, with each locus explaining between 3.7 to 34.9% of phenotypic variation^15–20^. Similar to *Fusarium* head blight (FHB), the development of black point-resistant wheat cultivars has been impeded because of poor understanding of the resistance mechanism^21^. Transcriptome analysis of wheat is a useful tool for understanding the resistance mechanism of wheat diseases, including leaf rust^22^, FHB^21^ and powdery mildew^23^.

In our previous study, we screened black point-resistant and -susceptible wheat lines^7^ based on the incidence under natural field condition and identified eight fungal species causing black point, including *Bipolaris sorokiniana*, *Alternaria alternata*, and *Fusarium equiseti*, of which *B. sorokiniana* was the most virulent pathogen^11^. Here, we used RNA-seq to conduct transcriptome analysis of slightly and highly susceptible kernels inoculated with *B. sorokiniana*. We also measured the activities of antioxidant enzymes, including superoxide dismutase (SOD), catalase (CAT), and peroxidase (POD). Our objectives were: (i) to analyze the major biological process associated with resistance to black point; and (ii) to assess whether the disease resistance mechanism is related to the ability to respond favorably to stress in wheat, including modulation of antioxidant enzyme activities.

## Results

### Symptom development among slightly and highly susceptible wheat lines

**Figure 1:**
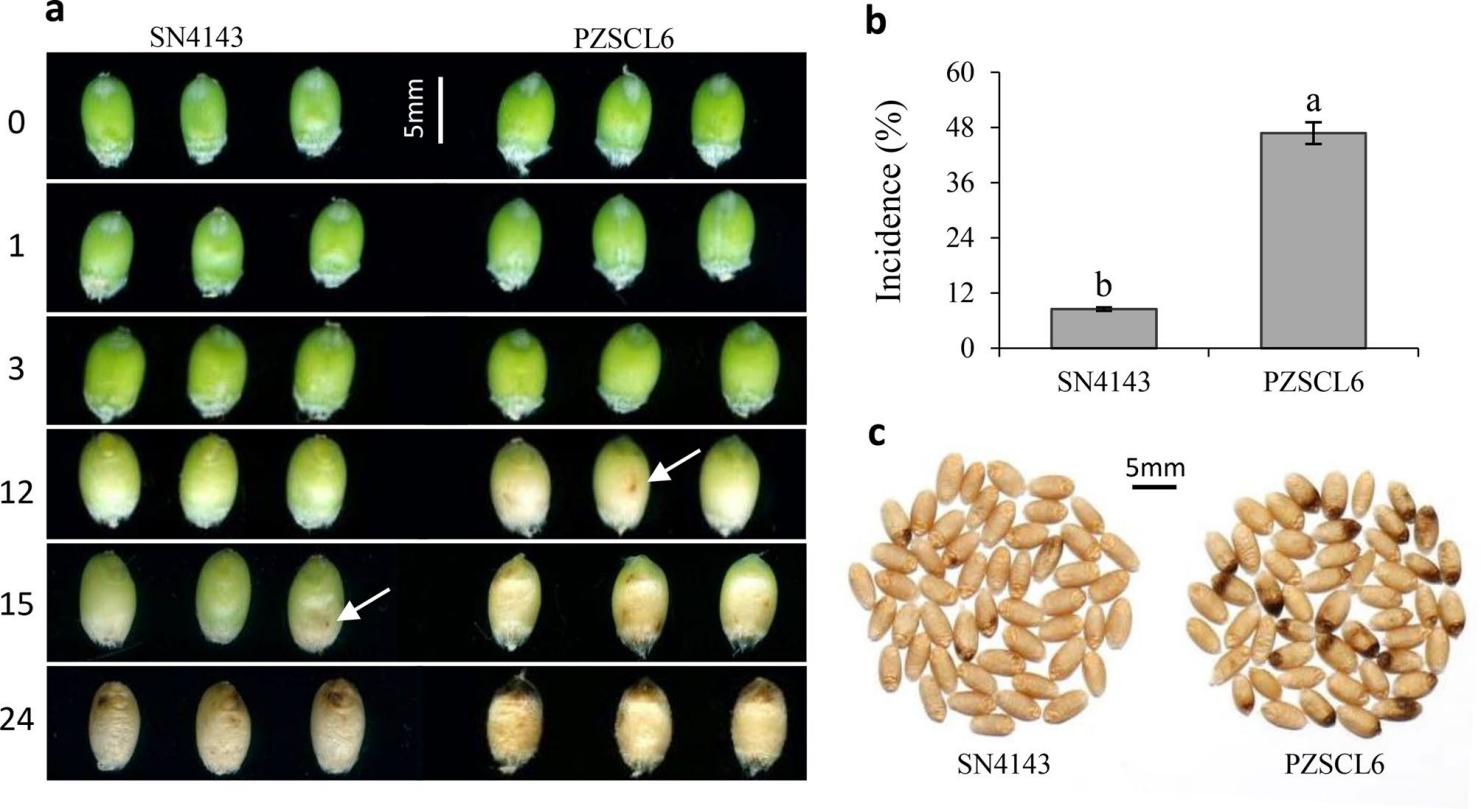
Symptom development of black point during grain filling stage (**a**) and mature kernels (**c**) from slightly susceptible SN4143 and highly susceptible PZSCL6 wheat line, and the incidence of SN4143 (8.5%) and PZSCL6 (46.8%) (**b**). 0, 1, 3, 12, 15 and 24 indicated the different days after inoculation. Bars (means) with different letters are significantly different (n = 3, *P* < 0.01).

The development of black point symptoms on kernels from the slightly susceptible SN4143 and highly susceptible PZSCL6 is shown in Fig. 1. A brown discoloration visible to the naked eye appeared on highly susceptible kernels at 12 days after inoculation with *B. sorokiniana*, and on slightly susceptible kernels at 15 days; at which time, the symptoms of black point on highly susceptible kernels were clearly visible. At 24 days after inoculation, the embryo and surrounding parts on kernels from the highly susceptible line developed brown or black discoloration, whereas the discolored area on kernels from the slightly susceptible line was smaller and lighter than that of the susceptible line (Fig. 1a).

The incidence of black point in the highly susceptible line was significantly greater than that in the slightly susceptible line (*P* < 0.01), with incidences of 46.8% and 8.5%, respectively (Fig. 1b,c). Moreover, the diseased kernels from the highly susceptible line displayed more serious symptoms than those of the samples from the slightly susceptible line (Fig. 1c). Symptom development in the slightly susceptible line was considerable slower, necessitating exploration of the mechanism of disease resistance.

### Overview of transcriptome sequencing data

**Figure 2:**
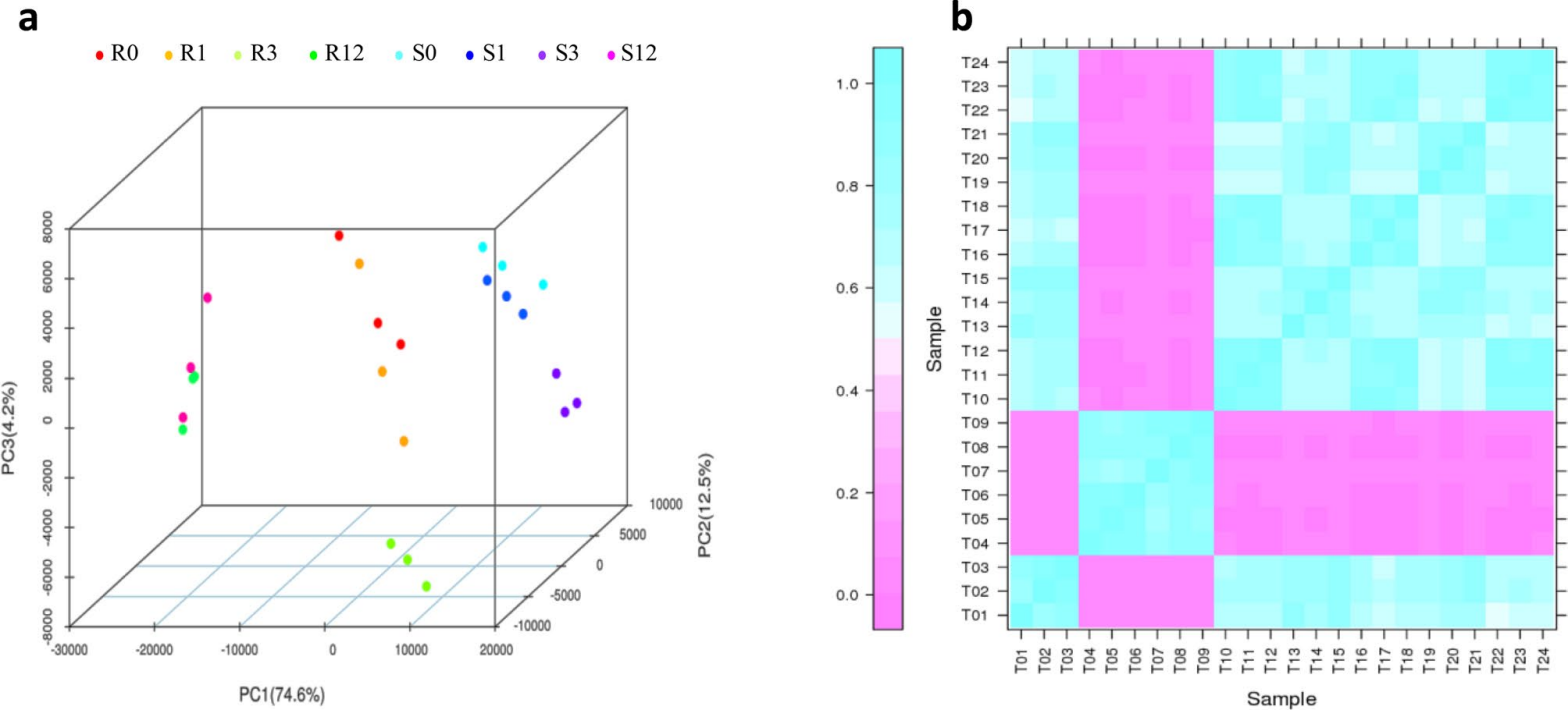
Correlations among the samples between slightly and highly susceptible lines at different timepoints. (**a**) Principal component analysis (PCA) map of the samples; (**b**) Pearson correlation coefficient (PCC) map of the samples. This figure was performed using BMKCloud (www.biocloud.net).

Twenty-four libraries divided amongst four groups of timepoints of 0, 1, 3, and 12 days were sequenced (R0: T1, T2, T3; R1: T13, T14, T15; R3: T19, T20, T21; R12: T4, T5, T6; S0: T10, T11, T12; S1: T16, T17, T18; S3: T22, T23, T24; S12: T7, T8, T9), and 251.8 Gb of clean bases obtained. The average Q30 percentage exceeded 92.3% (Supplementary Table S1). The comparison efficiency of reads in each sample compared with that of the *T. aestivum* reference genome was between 77.7% and 94.2%. The average comparison efficiency of the unique mapped reads was 78.0% (Supplementary Table S2). Principal component analysis (PCA) showed a good correlation among samples (Fig. 2a), and Pearson correlation coefficient (PCC) analysis showed that all correlation coefficients between replicate samples exceeded 0.81 (Fig. 2b), demonstrating high consistency of biological replicates.

**Figure 3:**
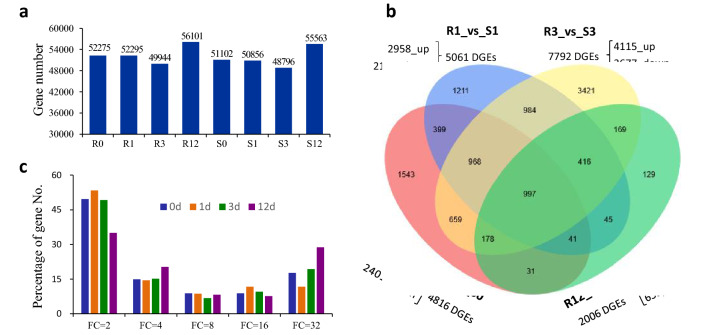
The differentially expressed genes (DEG) profiles. (**a**) Histogram of the gene numbers in the eight samples; (**b**) Venn diagram of DEG numbers in eight samples; (**c**) DEG numbers with different fold changes. 0, 1, 3, and 12 indicated the different days after inoculation. R and S are the slightly susceptible (SN4143) and highly susceptible (PZSCL6) line, respectively.

A total of 143,666 genes were identified in all samples, and each sample contained 89,631 genes (Supplementary Table S1). A total of 32,876 new genes were identified, of which 21,569 were functionally annotated with reference to the eight databases searched (Supplementary Table S3). We identified 55,275, 52,295, 49,944, 56,101, 51,102, 50,856, 48,796, and 55,563 genes (fragments per kilobase of transcript per million mapped fragments [FPKM] ≥ 0.5) from the eight samples R0, R1, R3, R12, S0, S1, S3, S12, respectively (Fig. 3a).

### Comparison of DEGs between black point-slightly and -highly susceptible lines at four timepoints

To explore the genes related to resistance to black point, differentially expressed genes (DEGs) were identified by pairwise comparisons of the 24 libraries. When fold change (FC, |Log2FC|= 2) and false discovery rate (FDR) < 0.01 were set as filtering thresholds, the number of DEGs identified among the four timepoint groups of R0 vs. S0, R1 vs. S1, R3 vs. S3, R12 vs. S12 were 4816, 5061, 7792, and 2006, respectively, at different fold changes (Fig. 3b, Supplementary Tables S4–S7). The number of DEGs decreased with a corresponding increase in the degree of fold change (Fig. 3c). The percentage of DEGs with a 32-fold change at 12 days after inoculation (R12 vs. S12) was the highest (28.8%), whereas that at 1 day (R1 vs. S1) was the lowest (11.7%).

The top metabolic pathways were classified based on gene annotations in the Kyoto Encyclopedia of Genes and Genomes (KEGG) database (Table 1). The top enriched pathways with DEGs of R0 vs. S0 were the same as R3 vs. S3 and are related to the process of photosynthesis (glyoxylate and dicarboxylate metabolism, photosynthesis-antenna proteins, and carbon fixation in photosynthetic organisms). The enriched pathways involving DEGs of R1 vs. S1 are also related to the process of photosynthesis, except for nucleotide excision repair. In contrast, the enriched pathways involving DEGs of R12 vs. S12 differed from those at days 1 to 3 and are principally involved in aminoacyl-tRNA biosynthesis, pyrimidine metabolism, and RNA transport.

**Table 1:** Major enrichment pathways of the DEGs from the four timepoint groups, referencing the KEGG database.

Sample pair	Pathway	Ko ID	*P*-value
R0d_vs_S0d	Glyoxylate and dicarboxylate metabolism	ko00630	2.46E−13
	Photosynthesis—antenna proteins	ko00196	2.75E−09
	Carbon fixation in photosynthetic organisms	ko00710	1.02E−08
R1d_vs_S1d	Carbon fixation in photosynthetic organisms	ko00710	5.21E−09
	Nucleotide excision repair	ko03420	2.15E−06
	Carbon metabolism	ko01200	3.76E−06
R3d_vs_S3d	Photosynthesis—antenna proteins	ko00196	< 1e−30
	Glyoxylate and dicarboxylate metabolism	ko00630	< 1e−30
	Carbon fixation in photosynthetic organisms	ko00710	< 1e−30
R12d_vs_S12d	Aminoacyl-tRNA biosynthesis	ko00970	4.49E−04
	Pyrimidine metabolism	ko00240	4.51E−03
	RNA transport	ko03013	1.53E−02

R and S are the slightly susceptible (SN4143) and highly susceptible (PZSCL6) line, respectively.

Further elaboration of the DEG analysis according to the gene number and *p*-value of the main terms in the Gene Ontology (GO) database are listed in Table 2, together with the top two enrichment terms of the DEGs for biological processes, cellular components, and molecular functions. All of the top enrichment items for biological processes at different days after inoculation were related to coping with stress (oxidation–reduction process, response to cadmium ion, and response to salt stress). The top enrichment terms for cellular components were cytoplasmic membrane-bounded vesicles, plasma membranes, and nucleosomes. For molecular function, the top terms were protein heterodimerization activity, peroxidase activity, and heme binding.

**Table 2:** Major enrichment terms of the DEGs from the four timepoint groups, referencing the GO database.

Sample pair	Class	Annotation	GO ID	*P*-value
R0d_vs_S0d	Biological process	Response to cadmium ion	GO:0046686	4.30E−16
		Response to salt stress	GO:0009651	1.30E−15
	Cellular component	Cytoplasmic membrane-bounded vesicle	GO:0016023	< 1e−30
		Plasma membrane	GO:0005886	1.90E−21
	Molecular function	Protein heterodimerization activity	GO:0046982	1.00E−19
		Peroxidase activity	GO:0004601	1.50E−14
R1d_vs_S1d	Biological process	Response to cadmium ion	GO:0046686	6.40E−16
		Response to salt stress	GO:0009651	7.00E−16
	Cellular component	Cytoplasmic membrane-bounded vesicle	GO:0016023	< 1e−30
		Nucleosome	GO:0000786	< 1e−30
	Molecular function	Protein heterodimerization activity	GO:0046982	9.40E−30
		Heme binding	GO:0020037	2.20E−14
R3d_vs_S3d	Biological process	Oxidation–reduction process	GO:0055114	1.80E−18
		Response to salt stress	GO:0009651	1.30E−15
	Cellular component	Cytoplasmic membrane-bounded vesicle	GO:0016023	< 1e−30
		Plasma membrane	GO:0005886	1.90E−21
	Molecular function	Peroxidase activity	GO:0004601	6.30E−16
		Heme binding	GO:0020037	1.60E−14
R12d_vs_S12d	Biological process	Response to salt stress	GO:0009651	2.10E−17
		Oxidation–reduction process	GO:0055114	1.60E−14
	Cellular component	Cytoplasmic membrane-bounded vesicle	GO:0016023	< 1e−30
		Plasma membrane	GO:0005886	4.20E−25
	Molecular function	Heme binding	GO:0020037	7.30E−16
		Peroxidase activity	GO:0004601	2.80E−15

R and S are the slightly susceptible (SN4143) and highly susceptible (PZSCL6) line, respectively.

### DEG co-expression clusters

**Figure 4:**
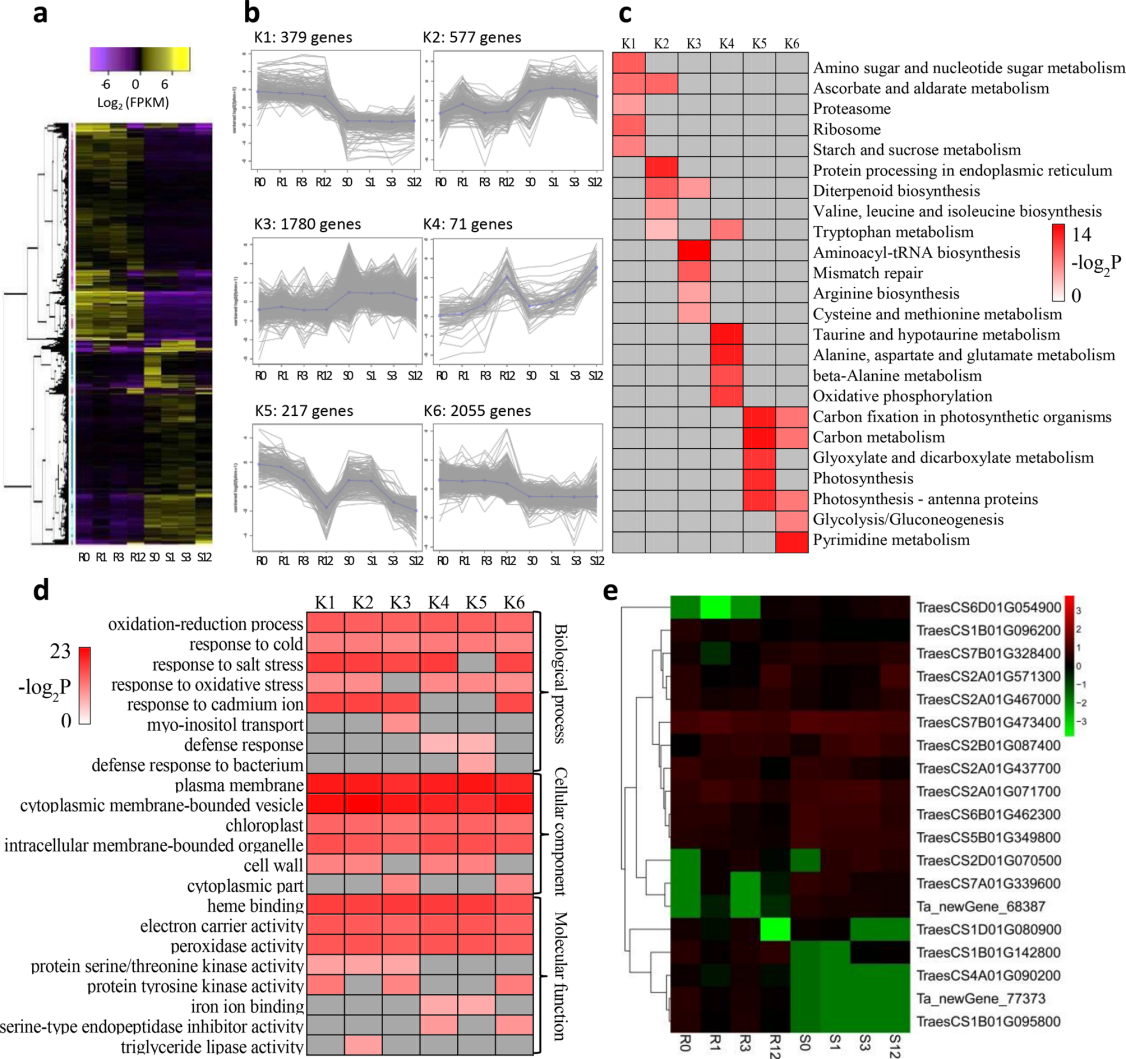
Overview of serial analysis of differentially expressed genes (DEGs) identified by pairwise comparisons between slightly and highly susceptible wheat lines at 0, 1, 3 and 12 days after inoculation with *Bipolaris sorokiniana*. (**a**) Heatmap of all DEGs among four timepoint samples for both lines. Expression values were presented as log_2_-transformed normalized FPKM values. (**b**) The six clusters (K1 to K6) for DEGs. (**c**) Top five enrichment pathways of the DEGs in different clusters, referring to the KEGG database. (**d**) Top five enrichment terms of the DEGs in different clusters, referring to GO database. The significance of the most represented pathways in each main cluster are indicated using -log_2_-transformed *P*-value (red); dark grey areas represent missing values. (**e**) Heat map of the 19 DEGs included in peroxidase activity (GO:0004601). R and S are the slightly susceptible (SN4143) and highly susceptible (PZSCL6) line, respectively. (**a,b,e**) were performed using BMKCloud (www.biocloud.net).

To discover gene expression trends from DEGs among the four timepoint groups, a gene co-expression analysis was performed using *k*-means clustering (*k* = 6). A total of 5079 genes (Supplementary Table S8) were selected and classified into six modules (Fig. 4a,b). Clusters K1 and K6, which included 2434 genes, were highly expressed in the slightly susceptible line compared with expression in the highly susceptible line. Genes in clusters K2 (577 genes) and K3 (1780 genes) were significantly expressed to a high level in the susceptible line. Genes in cluster K4 (71 genes) were also highly expressed in the susceptible line and, and unlike those in K2 and K3, continued to increase rapidly at 12 days after inoculation. The expression levels of genes in K5 in the slightly susceptible line were higher than those in the highly susceptible line at each sampling day; these differed from genes in clusters K1 and K6 and were highly expressed at day 0, although expression began to decrease at day 1, with gradually downregulation until 12 days after inoculation.

KEGG analysis was used to discover the metabolic pathways involved in the differentially clustered genes (Fig. 4c). For the six clusters, we discovered that many of the genes in clusters K1, K5, and K6 (all had upregulated expression in the slightly susceptible line) were involved in photosynthesis and sugar metabolism pathways, such as starch and sucrose metabolism (ko00500), carbon fixation in photosynthetic organisms (ko00710), carbon metabolism (ko01200), photosynthesis (ko00195), photosynthesis-antenna proteins (ko00196), and glycolysis/gluconeogenesis (ko00010). In contrast, many genes in clusters K2, K3, and K4 (where expression was downregulated in the slightly susceptible line) were involved in amino acid metabolism, such as valine, leucine, and isoleucine biosynthesis (ko00290), tryptophan metabolism (ko00380), arginine biosynthesis (ko00220), cysteine and methionine metabolism (ko00270), alanine, aspartate, and glutamate metabolism (ko00250); and beta-alanine metabolism (ko00410). These results indicate that photosynthesis-related metabolic pathways are important for the black point resistance processes caused by *B. sorokiniana*.

To explore the main terms associated with resistance to black point, the functions of DEGs in six clusters were classified based on the GO database; the top five terms of biological processes, cellular components and molecular functions are listed in Fig. 4d (*P* < 0.05). Genes included in different clusters were mainly involved in the biological processes of oxidation–reduction process (GO:0055114), response to cold (GO:0009409), salt stress (GO:0009651), oxidative stress (GO:0006979), and cadmium ion (GO:0046686). Genes concerning cellular components were mainly involved in plasma membrane (GO:0005886), cytoplasmic membrane-bounded vesicle (GO:0016023), chloroplast (GO:0009507), intracellular membrane-bounded organelle (GO:0043231), and cell wall (GO:0005618). Major molecular function-related genes were heme binding (GO:0020037), electron carrier activity (GO:0009055) and peroxidase activity (GO:0004601). These results indicate that resistance to black point was associated with the ability to reduce stresses caused by *B. sorokiniana*; integrity of the plasma membrane and intracellular membrane-bounded vesicles and organelles was important in this process. Further, electron carrier and peroxidase activities may also play important roles in coping with stresses caused by *B. sorokiniana* infection.

### Expression profiles of 12 DEGs

**Figure 5:**
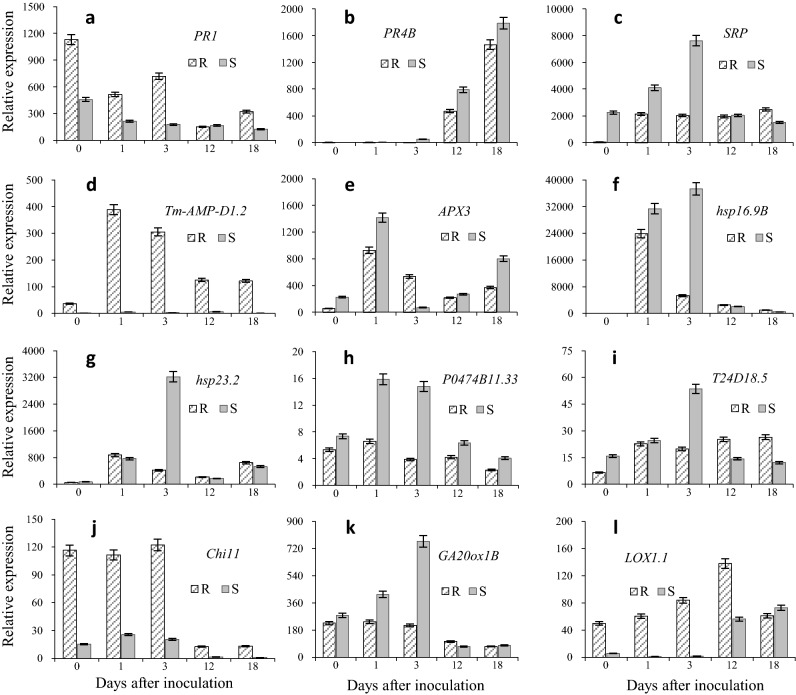
Spatiotemporal expression profiles of the twelve selected genes. (**a**) TraesCS2D01G317800 (pathogenesis-related protein 1); (**b**) TraesCS3D01G524700 (wheatwin-2, Precursor); (**c**) TraesCS2D01G114800 (stress-related protein); (**d**) TraesCS1A01G014000 (defensin Tm-AMP-D1.2); (**e**) TraesCS2A01G071700 (probable l-ascorbate peroxidase 3); (**f**) TraesCS3D01G045600 (16.9 kDa class I heat shock protein 2); (**g**) TraesCS2D01G311400 (23.2 kDa heat shock protein, Precursor); (**h**) TraesCS4D01G074600 (4-coumarate-CoA ligase-like 7); (**i**) TraesCS6D01G365700 (cinnamoyl-CoA reductase 1); (**j**) TraesCS5A01G500200 (chitinase 11, precursor); (**k**) TraesCS3B01G432800 (gibberellin 20 oxidase 1-B); (**l**) TraesCS4B01G037900 (linoleate 9S-lipoxygenase 1). The actin gene is used as internal control. The functional annotation and other details of A-L are listed in Table S9. All RT-qPCR reactions are replicated thrice. R and S are the slightly susceptible (SN4143) and highly susceptible (PZSCL6) line, respectively. Number 0, 1, 3, 6, 9, 12, 15, and 18 indicate the different days after inoculation.

To validate the reliability of the sequencing results, 12 important DEGs associated with stress or phenylpropanoid biosynthesis were selected from six clusters for assessment via reverse transcription quantitative PCR (RT-qPCR) (Fig. 5). These genes included: pathogenesis-related proteins (*PR1* and *PR4B*), stress-related protein (*SRP*), defensin (*Tm-AMP-D1.2*), l-ascorbate peroxidase 3 (*APX3*), heat shock proteins (*hsp16.9B* and *hsp23.2*), chitinase 11 (*Chi11*), gibberellin 20-oxidase 1-B (*GA20ox1B*), linoleate 9S-lipoxygenase 1 (*LOX1.1*), 4-coumarate-CoA ligase-like 7 (*P0474B11.33*), and cinnamoyl-CoA reductase 1 (*T24D18.5*). The RT-qPCR experimental samples were the same as those used for RNA-seq (except for samples at 18 days, which were only used for RT-qPCR). The results revealed that the expression changes of these 12 genes were consistent with the RNA-seq results (Supplementary Table S9).

The expression levels of *PR1*, *Tm-AMP-D1.2*, *Chi11*, and *LOX1.1* in the slightly susceptible line were higher than those in the highly susceptible line (Fig. 5a,d,j,l), although their expression profiles differed. For example, in the slightly susceptible line, *PR1* expression decreased on days 1 and 12, but increased on days 3 and 18; conversely, in the highly susceptible line, *PR1* expression gradually and consistently decreased (Fig. 5a). Expression of *Tm-AMP-D1.2* increased at day 1 and decreased gradually in the slightly susceptible line, whereas in the highly susceptible line, this was poorly expressed on all sampling days (Fig. 5d). As these four genes were all highly expressed in the slightly susceptible line, they may play an important role in the progression of resistance to black point caused by *B. sorokiniana*. Conversely, *hsp16.9B*, *hsp23.2*, *P0474B11.33*, and *GA20ox1B* were all more highly expressed in the highly susceptible line than in the slightly susceptible line. Expression of *P0474B11.33* increased at day 1 and then decreased at day 3 in both lines, although the expression level in the highly susceptible line was higher (Fig. 5h). In the slightly susceptible line, expression of *hsp16.9B*, *hsp23.2*, and *GA20ox1B* first increased and then decreased between days 1–3, although this continued to increase in the highly susceptible line over the same period (Fig. 5e–g,k). Thus, as these four genes were all highly expressed in the highly susceptible line, they may be involved in the pathogenic process of *B. sorokiniana*.

### Changes in antioxidant enzymes

**Figure 6:**
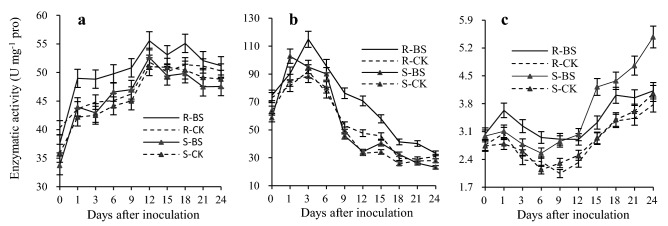
Activities of antioxidant enzymes in kernels from slightly and highly susceptible wheat lines inoculated with *Bipolaris sorokiniana*. (**a**) Superoxide dismutase (SOD); (**b**) catalase (CAT); (**c**) peroxidase (POD); The number 0, 1, 3, 6, 9, 12, 15, 18, 21 and 24 indicate the different days after inoculation. R and S are the slightly susceptible (SN4143) and highly susceptible (PZSCL6) line, respectively. Bs and CK represent the samples inoculated with *B. sorokiniana* and those treated with sterile distilled water, respectively.

Under infection conditions, plants activate a number of antioxidant enzymes that protect against potentially cytotoxic reactive oxygen species (ROS). Changes in the activities of antioxidant enzymes SOD, CAT, and POD are shown in Fig. 6. The activities of these enzymes in kernels from the slightly susceptible line increased significantly after inoculation with *B. sorokiniana*, except for POD, 12–24 days after inoculation.

There was a similar change in SOD activity in both lines after inoculation with *B. sorokiniana* (Fig. 6a). On all sampling days, the SOD activity of the slightly susceptible line was higher than that in the highly susceptible line. From day 1 to day 24 after inoculation, the SOD activity of the slightly susceptible line was 5.6% to 10.6% higher, with an average of 8.8%, whereas there was similar SOD activity of the CKs (treated with sterile distilled water) between the slightly and highly susceptible lines. The trend in CAT activity differed between the slightly and highly susceptible lines after infection (Fig. 6b). The maximal value of CAT activity was attained at day 1 (102.8 U mg^−1^ pro) in the highly susceptible line and at day 3 (115.0 U mg^−1^ pro) in the slightly susceptible line. In addition to the peak value, CAT activity of the slightly susceptible line was higher than that of the highly susceptible line; particularly on days 9 and 12, where the slightly susceptible line CAT activity was 64.6% and 76.9% higher, respectively, than that in the highly susceptible line. As with SOD activity, there was no clear difference in CAT activities between slightly and highly susceptible lines without *B. sorokiniana* inoculation. The change trend of POD activity was completely inconsistent between the two lines (Fig. 6c). On days 1–9 after inoculation, POD activity in the slightly susceptible line was higher than that in the highly susceptible line, whereas on days 12 to 24, this trend was reversed. In particular, at 24 days after inoculation, POD activity in the slightly susceptible line decreased, but that of the highly susceptible line continued to increase. The POD activity in the highly susceptible line was 26.8%, 9.1%, 20.4%, and 32.8% higher than that in the slightly susceptible line on days 15, 18, 21, and 24, respectively.

Hence, compared with that of the highly susceptible line, the measured activities of these three antioxidases were generally higher in the slightly susceptible line (except for POD at 12 to 24 days) following kernel inoculation with *B. sorokiniana*. Pathogen infection induced enhanced enzyme activities, which endowed the slightly susceptible line with a stronger oxidative stress response. However, expression of the 19 genes included in peroxidase activity (GO:0004601) was inconsistent between the two lines (Fig. 4e). For example, *PER2* (peroxidase 2 precursor) was highly expressed in the slightly susceptible line, whereas *PER70* (peroxidase 70 precursor) was highly expressed in the highly susceptible line.

## Discussion

RNA-seq is an effective method for studying the mechanisms of resistance to wheat diseases. For example, Li et al. used RNA-seq to identify DEGs in FHB-resistant and -susceptible wheat lines and detected an island of 53 constitutive DEGs in a 140 kb region on chromosome 3B^21^. Sharma et al. used a pair of near-isogenic lines (NILs) including HD 2329 (susceptible) and HD 2329 + *Lr28* (resistant) through RNA-seq to find DEGs associated with seedling leaf rust resistance mediated by the *Lr28* gene^22^. In the present study, we used RNA-seq to identify DEGs in wheat lines that were associated to resistance to black point at days 0, 1, 3, and 12 after inoculation with *B. sorokiniana*. According to the report of Li et al., the key periods and meteorological factors affecting black point are a humid environment at 1–15 days, lower temperature at 26–40 days, and a short period of sunshine after heading^14^. In mid to late April 2019 (about 1–15 days after heading), there are eight rain fall days, which was more than that in 2016 (6 days) and 2017 (1 day); the relative humidity was 60.6%, which was higher than that in 2016 (57.3%) and 2017 (41.9%)^14^. From May 1 to 20 (approximately 26–40 days after heading), average temperature was 22.1 ℃, which was between in 2016 (20.3 ℃) and 2017 (24.0 ℃). From April 14 to May 31, the daily sunshine duration was 6.4 h, which was between in 2016 (6.1 h) and 2017 (8.0 h)^14^. During the grain filling stage in 2019, there were no extreme meteorological factors affecting the wheat growth (Table S12). Compared with 2016 and 2017, the meteorological factors in 2019 were suitable for the development of black point. In addition, there is little difference in agronomic traits between the slightly susceptible SN4143 and highly susceptible PZSCL6 used in this study (Table S13). These results indicated that the significant difference in black point between SN4143 and PZSCL6 was probably due to their different resistance to *B. sorokiniana*.

### Photosynthesis is possibly involved in black point resistance

The plant’s response to pathogen attack is closely linked to a change in energy metabolic pathways such as the photosynthesis^21^. In the present study, we found that 73 DEGs involved in metabolic pathways related to photosynthesis, including carbon fixation in photosynthetic organisms, carbon metabolism, photosynthesis, photosynthesis-antenna proteins, glycolysis/gluconeogenesis, porphyrin and chlorophyll metabolism, was associated with black point resistance. Among the 73 DEGs involved in pathways related to photosynthesis, 58 (79.5%) had up-regulated expression in the samples from slightly susceptible wheat lines (Fig. 4c, Table S10). For example, FPKM values of the genes of Ribulose-1,5-bisphosphate carboxylase small subunit PWS 4.3 and PW9 (*TraesCS2B01G078900*, *TraesCS2A01G067300*), UDP-glucose/GDP-mannose dehydrogenase UGD5 (*Triticum_aestivum_newGene_28132*), Chlorophyll A-B binding protein (*TraesCS1B01G388200*, *TraesCS5B01G463000*) in the slightly susceptible samples was more than 10 times that of the susceptible samples. This result indicated that photosynthesis could play a vital role in regulating wheat resistance to black point, which was consistent with previous reports of wheat resistance to FHB^21,23^. Erayman et al. found similar numbers of transcripts involved in carbohydrate metabolism between moderately susceptible and susceptible wheat cultivars, whereas the number of down-regulated transcripts was higher in leaves of moderately susceptible cultivars than that in susceptible cultivars inoculated with *F. graminearum*^24^. Alterations in carbohydrate metabolism have been reported to occur during pathogen stress in wheat^25,26^. Reactions between host and pathogen trigger a multitude of genes in defense and energy production pathways^21,22,26^. As infection starts, the cells may require an increased level of energy production and the possible reasons may include the diversion of ATP usage towards gene expression and synthesis of defence-related proteins^23^. However, the detail of how carbohydrate metabolism regulates the resistance for wheat to black point caused by *B. sorokiniana* is unclear. Historically, immunity and photosynthesis have been studied separately and therefore discussing the cross-talk between these domains would be useful for understanding resistance mechanisms to black point.

### The ability to counteracting oxidative stress is related to black point resistance

Plants usually need to respond to different environmental changes and biotic stresses. Pathogens are a well-known example of biotic stress, and *B. sorokiniana* is particularly important because this can cause multiple diseases in wheat, including black point, root rot, and leaf spot^4,11,27^. Therefore, plants have evolved complex strategies to allow rapid modulation of biological processes and cellular functions of an active defense response, such as increasing antioxidase activity and inducing the expression of various genes related to oxidative stress, including genes of pathogenesis-related (PR) proteins^26–28^. In general, the major ROS-scavenging enzyme pathways of plants include SOD, CAT, and POD, the ascorbate–glutathione cycle, and the glutathione peroxidase cycle^28^. When plants are infected by fungi, the activities of antioxidant enzymes are increased to counteract oxidative stress. For example, the activities of CAT, POD, and ascorbate peroxidase (APX) increased after inoculation with *Fusarium* species^29^. In the present study, GO analysis of DEGs following *B. sorokiniana* infection indicated the enrichment of transcripts mainly involved in redox processes (response to cold, salt stress, oxidative stress, and cadmium ion) and the activities of SOD, POD, and CAT all increased following inoculation. Compared with that of the highly susceptible line, the measured activities of these three antioxidases were generally higher in the slightly susceptible line (except for POD at 12 to 24 days).

Pathogenesis-related (PR) proteins such as chitinases, glucanases and defensins are important to improve resistance to fungal diseases in many plants, including wheat. In the current study, 64 DEGs associated with PR protein was detected, including *PR-1*, *PR-2*, *PR-3*, *PR-4*, *PR-5*, *PR-6*, *PR-12*, *PR-13*, *PR-14* and *PR-15*, among which, 47 (73.4%) had up-regulated expression in the slightly susceptible kernels, especially three *PR* genes (*PR1*, *Tm-AMP-D1.2* and *Chi11*) validated via RT-qPCR (Fig. 5, Table S10). Defensing, including Tm-AMP-D1.2, is the most important antimicrobial peptide family involved in defense functions and is postulated to be involved in defense against fungal and bacterial pathogens and insect pests^30,31^. Chitinase genes (*Chi*) have also been reported to play important roles in defense reactions. Ghorbel et al. (2020) isolated a novel PR gene (*TdPR1.2*) from a durum wheat variety that exhibits an antibacterial effect against eight different bacteria and the fungi *Septoria tritici*^32^. In another study, the expression of *PR1.2* and *Chi-1* was significantly increased in the resistant wheat cultivar after inoculation with *F. graminearum*^33^. Future studies will therefore investigate and focus on the role of these genes in resistance to black point.

Numerous studies have reported that symptoms of black point result from enzymatic browning^34–37^. Under normal growth conditions, the production of blackening substances that cause black point symptoms is not evident even though plants normally contain both the enzymes (POD and polyphenol oxidase) and the substrates (various phenolic compounds) for enzymatic browning. The suggested reasons for lack of reaction mainly include: (i) compartmentalization of enzyme and substrate in different parts of the cell^38,39^, and (ii) the enzyme is present in an inactive form requiring activation^39,40^. Under the inoculation condition, a failure to effectively eliminate excessive ROS can cause protein and lipid peroxidation or even cell death^41^. This study showed that activity of antioxidant enzymes, including SOD, CAT, and POD, were increased by inoculation with *B. sorokiniana* to respond with oxidative stress in wheat kernels. The activities of the antioxidant enzymes in the slightly susceptible line were higher than those in the highly susceptible line, so the excessive ROS caused by *B. sorokiniana* were eliminated more efficiently in the slightly susceptible line than that in the highly susceptible line during the kernel filling stage. Strong antioxidant capacity associated with resistance mechanism to black point, also been reported in other studies. Mak et al. used proteomic analysis to identify the proteins from black point-affected and -free grains of the susceptible wheat cultivar SUN239V. They found that of 12 functional classes to which the differentially abundant proteins were assigned the largest was the ‘stress’ class (i.e., gene products of genes associated with stress, disease and defense)^10^. Higher levels of these ‘stress’ proteins were found in black point-free grains, suggesting that increased levels of these proteins might afford protection from the disease, which was consistent with the higher expression of PR genes in slightly susceptible kernels in the current study. Therefore, we suggest that the membrane system integrity of the susceptible line was destroyed because the excessive ROS could not be eliminated quickly; this was reflected by the higher malondialdehyde content in the susceptible line^42^, which led to the substrates in contact with the enzyme of enzymatic browning to produce blackening substances. This result is also supported by our GO analysis, which revealed enrichment in cellular component terms associated with the plasma membrane and cytoplasmic membrane-bound vesicles (Fig. 5d).

Some candidate genes for black point resistance have been identified through BLAST against the National Center for Biotechnology Information (NCBI, http://www.ncbi.nlm.nih.gov/) and European Nucleotide Archive (ENA, http://www.ebi.ac.uk/ena) databases, using the flanking sequences of SNP markers significantly associated with black point reaction as queries^16,17,19^. These candidate genes could be divided into different groups. For example, genes related to (i) enzymes needed in enzymatic browning, including POD and PPO; (ii) the signal transduction pathways of plant hormones, including gibberellin biosynthetic genes, F-box repeat and serine/threonine-protein kinase and its receptor; (iii) defense mechanisms during stress response in plants, including zinc finger proteins, disease resistance *RPP8*-like protein, U-box domain-containing protein, and MYB transcription factor^16,17,19^, which was supported by results of the current study. There were 19, 3, 15, 81, 68, 137, 7, 13, and 31 genes related to POD, PPO, gibberellin biosynthesis, F-box protein, serine/threonine-protein kinase and associated receptor, zinc finger protein, *RPP8*-like protein, U-box domain-containing protein, and MYB transcription factor, respectively, which were differentially expressed between the slightly and highly susceptible wheat lines after inoculation with *B. sorokiniana* (Fig. 4e, Supplementary Table S10).

Here, we describe that resistance to *B. sorokiniana*-caused black point in wheat was predominately related to the plant counteracting oxidative stress. However, the biological processes and metabolic pathways involved in coping with oxidative stress in plants is complicated, which is indicated by the variety of GO and KEGG enrichment terms uncovered in this study. Thus, more detailed experimental analyses are required to confirm the specific involvement of specific metabolic pathways in resistance to black point, using more suitable materials such as mutants and NILs.

## Materials and methods

### Plant materials and field management

Two wheat lines**,** SN4143 and PZSCL6, were screened from 403 wheat genotypes, which was resistant and susceptible under the natural field condition in 2010–2012^7^. The seeds were provided by Henan Academy of Agricultural Science, China. The average incidence of black point in SN4143 and PZSCL6 was 46.8% (highly susceptible) and 6.75% (slightly susceptible), respectively, under conditions of inoculation with *B. sorokiniana* from 2016 to 2017 in Henan Province, China. Twenty-one major resistant marker-trait associations were identified by genome-wide association study in line SN4143, whereas 11 were identified in the susceptible lines PZSCL6^19^. These two wheat lines were planted in six 2 m long rows spaced 20 cm apart (50 seeds per row) during the 2018–2019 season in an experimental field of Henan Agricultural University (113° 42′ E, 34° 44′ N), Zhengzhou, Henan Province, China. Seeds were planted in the middle of October and harvested at the beginning of June. The experiment was repeated three times.

### Symptom induction of the black point

Fungal isolate Ta-BP33, representing *B. sorokiniana*^11^, was selected as the experimental pathogen. Ta-BP33 was cultured on potato dextrose agar medium in a 9-cm Petri dish and incubated for 10 to 12 days in a dark growth chamber at 25 ± 1 °C. Conidial suspensions were prepared as in Mahto et al.^43^, and the concentration was standardized to 3 × 10^5^ conidia per mL. Inoculation was performed using a previously reported method^44^. Briefly, at Zadoks growth stage GS 55^45^, 150 spikes from each wheat line of each replicate were covered with sulfuric acid-paper bags (five spikes per bag) to prevent contamination. Subsequently, at GS 65, spikes were inoculated with conidial suspensions using a hand sprayer until they were dripping (the control was treated with sterile distilled water) and were then covered with transparent plastic bags for 5 days to maintain humidity.

### Sample preparation and RNA extraction

At 0, 1, 3, 6, 9, 12, 15, 18, 21, and 24 days after inoculation, kernels inoculated with *B. sorokiniana* from both lines were selected, frozen immediately in liquid nitrogen, and stored at − 80 °C. Bulk samples at different days after inoculation from both lines (designated R0, R1, R3, R6, R9, R12, R15, R18, R21, and R24 for the slightly susceptible line and S0, S1, S3, S6, S9, S12, S15, S18, S21, and S24 for the highly susceptible line) were prepared with three biological replicates for a total of 60 samples. Each bulk included 15 independent individuals. Samples of 0, 1, 3, and 12 days (24 samples) were used for transcriptome sequencing, those of 0, 1, 3, 12, and 18 days (30 samples) were used for RT-qPCR, and all 60 samples were used for analysis of enzyme activity and observation of symptom development of black point.

Total RNA was extracted as described by Li et al.^21^. DNA was removed with DNase treatment (Invitrogen, Shanghai, China). RNA concentration and integrity were measured using a NanoDrop 2000 (NanoDrop Technologies, Wilmington, DE, USA) and an RNA Nano 6000 Assay Kit (Agilent Technologies, CA, USA), respectively.

### Transcriptome sequencing and data analysis

Twenty-four mRNA libraries were constructed, sequenced, and clean reads obtained according to the procedure of Li et al.^46^. The clean reads were mapped to the reference wheat genome version, IWGSC_RefSeq_v1.0. (https://urgi.versailles.inra.fr/download/iwgsc/IWGSC_RefSeq_Assemblies/v1.0/).

Annotation of the transcriptome sequences was performed using homologous sequences obtained by BLAST searching^47^ against eight public databases: Non-Redundant Protein; Swiss-Prot; GO; Cluster of Orthologous Genes; Eukaryotic Orthologous Groups; Protein Family; Evolutionary Genealogy of Genes: Non-supervised Orthologous Group; and KEGG. The biological pathways (with reference to the KEGG database) of the new genes were analyzed using KOBAS2.0^48^ and their amino acid sequences were predicted and annotated using HMMER^49^. Quantitative gene expression was calculated using FPKM^50^.

Pairwise difference analysis was conducted on gene expression levels among the four groups using DESeq R packages^51^. DEGs were identified by FC (|Log2FC|≥ 1) and FDR ≤ 0.01 parameters. PCC and PCA were performed to evaluate the indices of biological repetition correlation^52,53^. A |Log2FC|≥ 2 was chosen as the threshold value for performing gene co-expression analysis by K-means clustering^46,54^. All transcriptome data analyses used BMKCloud (www.biocloud.net). The BioProject ID of the transcriptome sequence data in NCBI is PRJNA664832.

### RT-qPCR

Samples from both lines at five time points (0, 1, 3, 12, and 18 days after inoculation) were prepared for RT-qPCR. All primers were designed using Premier 5.0 (http://www.premierbiosoft.com/primerdesign/index.html; primer information is shown in Supplementary Table S11). Reverse transcription was carried out as described by Li et al.^46^. RT-qPCR reactions were carried out in a total volume of 20 μL, with the actin gene used as an internal control. Gene expression levels were calculated according to the 2^−ΔΔCT^ method^55^. All RT-qPCR reactions are replicated thrice.

### Determination of enzyme activity

Wheat kernels (0.5 g) were homogenized in 5 mL of phosphate buffer at pH 7.6 for SOD, pH 6.0 for POD, and pH 6.8 for CAT. Enzyme activities were measured spectrophotometrically (UV-5200, Metash Instruments Co. Ltd, Shanghai, China) by specific methods. SOD activity was estimated as the inhibition of photochemical reduction of nitroblue tetrazolium (NBT) at 560 nm, according to Beauchamp and Fridovich^56^. One unit of SOD was equivalent to the enzyme concentration that caused 50% photoreduction of NBT. POD activity was estimated as described by Zhang and Qu^57^. The reaction mixture contained phosphate buffer (pH 6.0), guaiacol (1.25%, v/v), and 0.1 mM H_2_O_2_, and activity was determined by measuring the oxidation products of guaiacol (extinction coefficient, 26.6 mM^−1^ cm^−1^) at 470 nm. Enzyme activity was calculated as µmol oxidation products formed mg^−1^ protein min^−1^. CAT activity was detected via the method of Cakmak and Horst^58^. The reaction mixture (3 mL) contained 1.5 mL of phosphate buffer (pH 6.8) and 1 mL of 0.2% H_2_O_2._ CAT activity was calculated as µmol H_2_O_2_ (extinction coefficient, 39.4 mM^−1^ cm^−1^) decomposed mg^−1^ protein min^−1^.

### Statement of ethical standards

The two wheat lines provided by Henan Academy of Agricultural Sciences in the experiment are the materials we have been using for many years in our breeding. We declare that these experiments comply with the ethical standards in China.

## Conclusions

Here, we used comparative transcriptome analysis to identify DEGs in black point -slightly and -highly susceptible wheat lines inoculated with *B. sorokiniana.* We determined the top GO database enrichment terms for biological processes as oxidation–reduction process, response to cold, salt stress, oxidative stress, and cadmium ion. Moreover, we showed that the activities of antioxidant enzymes, including SOD, CAT, and POD, were higher in the slightly susceptible lines than those in the highly susceptible lines. These results indicate that resistance to *B. sorokiniana*-induced black point in wheat was predominately related to the ability to respond to oxidative stress. This study presents new insights for understanding the resistance mechanisms of selected wheat lines to black point.

## Supplementary Information

Supplementary Information 1.

Supplementary Information 2.

## References

[1] Statler GD, Kiesling RL, Bosch RH (1975). Inheritance of black point resistance in durum wheat. Phytopathology.

[2] Solanki VA, Augustine N, Patel AA (2006). Impact of black point on wheat trade and its management. Indian Phytopathol..

[3] El-Gremi SM, Draz IS, Youssef WAE (2017). Biological control of pathogens associated with kernel black point disease of wheat. Crop Protect..

[4] Li QY (2020). Assessing genetic resistance in wheat to black point caused by six fungal species in the Yellow and Huai wheat area of China. Plant Dis..

[5] Conner RL, Davidson JGN (1988). Resistance in wheat to black point caused by *Alternaria alternata* and *Cochliobolus sativus*. Can. J. Plant Pathol..

[6] Rees RG, Martin DJ, Law DP (1984). Black point in bread wheat. Effects on quality and germination and fungal associations. Aust. J. Exp. Agric. Anim. Husb..

[7] Li QY (2014). Screening wheat genotypes for resistance to black point and the effects of diseased kernels on seed germination. J. Plant Dis. Protect..

[8] Palacios SA (2015). Genetic variability and fumonisin production by *Fusarium proliferatum* isolated from durum wheat grains in Argentina. Int. J. Food Microbiol..

[9] Masiello M (2020). Molecular identification and mycotoxin production by *Alternaria* species occurring on durum wheat, showing black point symptoms. Toxins.

[10] Mak Y (2006). Black point is associated with reduced levels of stress, disease- and defence-related proteins in wheat grain. Mol. Plant Pathol..

[11] Xu KG (2018). Identification and pathogenicity of fungal pathogens causing black point in wheat on the North China Plain. Indian J. Microbiol..

[12] Somma S, Amatulli MT, Masiello M, Moretti A, Logrieco AF (2019). *Alternaria* species associated to wheat black point identified through a multilocus sequence approach. Int. J. Food Microbiol..

[13] Sisterna MN, Sarandon SJ (2005). Preliminary studies on the natural incidence of wheat black point under different fertilization levels and tillage systems in Argentina. Plant Pathol. J..

[14] Li QY (2019). Key periods and effects of meteorological factors affecting incidence of wheat black point in the Yellow and Huai wheat area of China. Crop Protect..

[15] Lehmensiek A (2004). QTLs for black-point resistance in wheat and the identification of potential markers for use in breeding programs. Plant Breed..

[16] Liu JD (2016). Genome wide linkage mapping of QTL for black point reaction in bread wheat (*Triticum aestivum* L.). Theor. Appl. Genet..

[17] Liu JD (2017). Genome-wide association mapping of black point reaction in common wheat (*Triticum aestivum* L.). BMC Plant Biol..

[18] Lv GG (2020). Identification of genetic loci of black point in Chinese common wheat by genome-wide association study and linkage mapping. Plant Dis..

[19] Li QY (2020). GWAS for resistance against black point caused by *Bipolaris sorokiniana* in wheat. J. Cereal Sci..

[20] Wang SY (2020). Genetic analysis and detection of resistance loci for black point in wheat genotype Shannong. Acta Phytopathol. Sin..

[21] Li X (2018). Transcriptome analysis identifies a 140 kb region of chromosome 3B containing genes specific to *Fusarium* head blight resistance in wheat. Int. J. Mol. Sci..

[22] Sharma C (2018). A study of transcriptome in leaf rust infected bread wheat involving seedling resistance gene *Lr28*. Funct. Plant Biol..

[23] Hill-Ambroz K (2006). Expression analysis and physical mapping of a CDNA library of Fusarium head blight infected wheat spikes. Crop Sci..

[24] Erayman M (2015). Transcriptome analysis of wheat inoculated with *Fusarium graminearum*. Front. Plant Sci..

[25] Wright D, Baldwin B, Shephard M, Scholes J (1995). Source-sink relationships in wheat leaves infected with powdery mildew. I. Alterations in carbohydrate metabolism. Physiol. Mol. Plant Pathol..

[26] Kumar S (2014). *Lr1*-mediated leaf rust resistance pathways of transgenic wheat lines revealed by a gene expression study using the Affymetrix GeneChip wheat genome array. Mol. Breed..

[27] Ye W (2019). Disclosure of the molecular mechanism of wheat leaf spot disease caused by *Bipolaris sorokiniana* through comparative transcriptome and metabolomics analysis. Int. J. Mol. Sci..

[28] Mittler R (2002). Oxidative stress, antioxidants and stress tolerance. Trends Plant Sci..

[29] Gherbawy Y, El-Tayeb M, Maghraby T, Shebany Y, El-Deeb B (2012). Response of antioxidant enzymes and some metabolic activities in wheat to *Fusarium* spp.. Acta Agron. Hung..

[30] Odintsova TI (2019). Defensin-like peptides in wheat analyzed by whole-transcriptome sequencing: A focus on structural diversity and role in induced resistance. PeerJ.

[31] Vriens K, Cammue BPA, Thevissen K (2014). Antifungal plant defensins: mechanisms of action and production. Molecules.

[32] Ghorbel M (2020). Differential regulation of the durum wheat Pathogenesis-related protein (PR1) by Calmodulin TdCaM1.3 protein. Mol. Boil. Rep..

[33] Soltanloo H (2010). The expression profile of *Chi-1*, *Glu-2*, *Glu-3* and *PR1.2* genes in Scab-resistant and susceptible wheat cultivars during infection by *Fusarium graminearum*. Plant Omics J..

[34] Williamson PM (1997). Black point of wheat in vitro production of symptoms, enzymes involved, and association with *Alternaria alternata*. Aust. J. Agric. Res..

[35] Walker JRL, Ferrar PH (1998). Diphenol oxidase, enzyme-catalysed browning and plant disease resistance. Biotechnol. Genet. Eng..

[36] Walker, K. R. Regulation of candidate genes in black point formation in barley. Doctoral Dissertation, the University of Adelaide, Adelaide, Australia (2011)*.*

[37] Li QY (2020). Enzymatic browning in wheat kernels produces symptom of black point caused by *Bipolaris sorokiniana*. Front. Microbiol..

[38] Mayer AM, Andharel E (1979). Polyphenol oxidases in plants. Phytochemistry.

[39] Vaughn KC, Duke SO (1984). Function of polyphenol oxidase in higher plants. Physiol. Plant..

[40] Vámos-Vigyázó L, Haard NF (1981). Polyphenol oxidase and peroxidase in fruits and vegetables. Crit. Rev. Food Nutr..

[41] Deslile G, Champoux M, Houde M (2001). Characterization of oxalate oxidase and cell death in Al-sensitive and tolerant wheat roots. Plant Cell Physiol..

[42] Wang J (2007). Study on the PPO activity, content of MDA and phenolics in wheat ears with different resistance to black point after inoculation with *Alternaria alternate*. Henan Sci..

[43] Mahto BN, Gurung S, Adhikari TB (2011). Assessing genetic resistance to spot blotch, *Stagonospora nodorum* blotch and tan spot in wheat from Nepal. Eur. J. Plant Pathol..

[44] Li QY (2019). A method for joint identification of wheat resistance to black point and leaf blight disease caused by *Bipolaris sorokiniana*, ZL2015105396457.

[45] Zadoks J, Chang T, Konzak C (1974). A decimal code for the growth stages of cereals. Weed Res..

[46] Li JC (2019). Gene expression profiles and microRNA regulation networks in tiller primordia, stem tips, and young spikes of wheat Guomai 301. Genes.

[47] Altschul SF (1997). Gapped BLAST and PSI BLAST: A new generation of protein database search programs. Nucleic Acids Res..

[48] Xie C (2011). KOBAS 2.0: A web server for annotation and identification of enricheded pathways and diseases. Nucleic Acids Res..

[49] Eddy SR (1988). Profile hidden Markov models. Bioinformatics.

[50] Florea L, Song L, Salzberg SL (2013). Thousands of exon skipping events differentiate among splicing patterns in sixteen human tissues. F1000 Res..

[51] Anders S, Huber W (2011). Differential expression analysis for sequence count data. Genome Biol..

[52] Pearson E (1931). The test of significance for the correlation coefficient. J. Am. Stat. Assoc..

[53] Zou H, Hastie T, Tibshirani R (2006). Sparse principal component analysis. J. Comput. Graph. Stat..

[54] Hartigan JA, Wong MA, Algorithm AS (1979). A K-means clustering algorithm: Algorithm as 136. Appl. Stat..

[55] Livak KJ, Schmittgen TD (2001). Analysis of relative gene expression data using real-time quantitative PCR and the 2^−ΔΔCT^ method. Methods.

[56] Beauchamp C, Fridovich I (1971). Superoxide dismutase: Improved assays and applicable to acrylamide gels. Anal. Biochem..

[57] Zhang ZL, Qu WJ (2003). Experimental Guidance of Plant Physiology.

[58] Cakmak I, Horst WJ (1991). Effect of aluminum on lipid peroxidation, superoxide dismutase, catalase, and peroxides activities in root tips of soybean (*Glycine max*). Physiol. Plant..

